# Ultrasmall episymbiont *Nanosynbacter lyticus* employs multiple ATP-generating metabolic pathways during horizontal transmission

**DOI:** 10.1093/ismejo/wraf288

**Published:** 2025-12-29

**Authors:** Nusrat Nahar, Pu-Ting Dong, Jing Tian, Alex S Grossman, Erik L Hendrickson, Kristopher A Kerns, Mary Ellen Davey, Batbileg Bor, Jeffrey S McLean, Xuesong He

**Affiliations:** Department of Microbiology, ADA Forsyth Institute, Somerville, MA 02143, United States; Department of Microbiology, ADA Forsyth Institute, Somerville, MA 02143, United States; Department of Pediatric Dentistry, Peking University School and Hospital of Stomatology, Beijing, Beijing Municipality 100081, China; Department of Microbiology, ADA Forsyth Institute, Somerville, MA 02143, United States; Department of Periodontics, University of Washington, Seattle, WA 98195, United States; Department of Periodontics, University of Washington, Seattle, WA 98195, United States; Department of Microbiology, ADA Forsyth Institute, Somerville, MA 02143, United States; Department of Microbiology, ADA Forsyth Institute, Somerville, MA 02143, United States; Department of Periodontics, University of Washington, Seattle, WA 98195, United States; Department of Microbiology, University of Washington, Seattle, WA 98109, United States; Department of Oral Health Sciences, University of Washington, Seattle, WA 98195, United States; Department of Microbiology, ADA Forsyth Institute, Somerville, MA 02143, United States

**Keywords:** *Saccharibacteria*TM7, patescibacteria, candidate phyla radiation, episymbiont, arginine deiminase system (ADS), glycolysis

## Abstract

*Saccharibacteria* (formerly TM7) are a group of environmentally diverse, ultrasmall bacteria with highly reduced genomes belonging to Patescibacteria (formerly Candidate Phyla Radiation), a newly identified bacterial lineage accounting for over a quarter of microbial diversity. *Nanosynbacter lyticus* strain TM7x was isolated from the human oral cavity and was the first culture representative of *Saccharibacteria*. It displays an obligate episymbiotic lifestyle where TM7x lives on the surface of its bacterial host *Schaalia odontolytica* strain XH001. *Saccharibacteria* rely on host bacteria for growth. TM7x multiplies through budding division, and daughter cells can disassociate from host bacteria during their horizontal transmission stage and establish symbiosis with new bacterial hosts. However, how these metabolically constrained symbionts maintain their viability and infectivity during their horizontal transmission phase, when they are disassociated from hosts, remains poorly understood. By applying targeted mutagenesis using recently developed genetic tools for *Saccharibacteria*, we demonstrate that the TM7x-encoded arginine deiminase system (ADS) plays a critical role in ATP production and impacts TM7x-host bacterium interaction. Furthermore, we present the first empirical evidence showing that TM7x can uptake and utilize glucose via the glycolysis pathway. Glycolysis is particularly important for episymbiont ATP production under anoxic conditions during horizontal transmission between hosts. Our study demonstrates that TM7x employs two ATP-generating metabolic pathways, ADS and glycolysis, to ensure its viability and infectivity under different microenvironments when disassociated from its hosts during horizontal transmission, a critical phase of its life cycle.

## Introduction

Patescibacteria, also known as the Candidate Phyla Radiation, represents one of the largest and most enigmatic branches of the bacterial domain, comprising lineages with reduced genomes and limited biosynthetic capabilities [1–4]. All the Patescibacteria that have been cultivated to date are symbiotic, relying on physical and metabolic associations with host organisms to survive [5]. Among these taxa, *Saccharibacteria* (formerly TM7) are notable for their obligate episymbiotic lifestyles [5–8] as well as their widespread presence in the environment and mammalian body sites, including the human oral cavity [4]. Despite their ubiquity, the biology of *Saccharibacteria* remains poorly understood due to long-standing challenges in cultivation and a lack of genetic tools.

*Nanosynbacter lyticus* strain TM7x, the first successfully cultured *Saccharibacteria*, was isolated from the human oral cavity and forms an obligate episymbiotic relationship with its host, *Schaalia odontolytica* strain XH001 [5]. TM7x attaches to the surface of XH001, replicates via budding, and relies on the host bacteria for essential nutrients and metabolic support [5, 9]. During its life cycle, TM7x enters a horizontal transmission phase, in which daughter cells transiently detach from the host bacteria before establishing new infections [5, 10, 11], however, these associations are typically limited to closely related *Schaalia* species [12]. This free-floating stage poses a critical physiological challenge for TM7x due to its streamlined genome (~705 kb) that lacks most canonical de novo biosynthetic pathways and central metabolism [3, 5]. How TM7x remains viable and maintains infectivity during this stage is not well understood.

Despite its reduced genome, TM7x encodes a complete arginine deiminase system (ADS), a common energy-yielding pathway that is widely distributed among prokaryotes and plays important roles in acid resistance, anoxic ATP production, and interspecies competition [10, 13]. Recent studies suggest that only mammalian-associated *Saccharibacteria* have acquired the ADS, likely during their transition from environmental to mammalian niches [10], underscoring its potential importance for adaptation and survival within mammalian microbiomes. ADS converts arginine to ornithine, ammonia, and ATP via three key enzymes: arginine deiminase (*arcA*), ornithine transcarbamylase (*arcB*), and carbamate kinase (*arcC*) [10, 14, 15]. TM7x also encodes the arginine transporter, *arcE*, that allows extracellular arginine uptake. Our previous study provided experimental evidence suggesting that TM7x-encoded ADS is functional and plays important roles in ATP production and acid tolerance [10]. However, due to the lack of a genetic system, direct evidence from targeted gene knockout remains unavailable.

Genomic analyses show that despite lacking a complete tricarboxylic acid cycle, many *Saccharibacteria*, including TM7x, may possess limited fermentative capacity as evidenced by the presence of glycolysis-associated genes [11, 16, 17]. Recent work has revealed that some Patescibacteria, including *Saccharibacteria*, may retain glycolytic enzymes to scavenge sugars from host or environmental sources [17]. Yet, whether TM7x can metabolize glucose for energy generation, particularly during its free-floating stage, to maintain viability and infectivity, has not been experimentally demonstrated.

In this study, we employed genetic and functional approaches to dissect the ATP-generating strategies of TM7x. Using targeted gene knockouts of *arcA* and *arcE*, we demonstrate that the TM7x-encoded ADS is critical for ATP production and contributes to the efficiency of host attachment. Moreover, we provide the first experimental evidence that TM7x can uptake and metabolize glucose via glycolysis, particularly under anoxic conditions. Together, these results shed light on the metabolic flexibility of *Saccharibacteria* and suggest mechanisms by which these episymbionts survive free-floating periods and maintain high prevalence in host-associated microbiomes.

## Results

### Deletion of *arc* genes impairs ADS functionality and ATP production

The ADS is a key energy-generating pathway allowing bacteria to utilize arginine and produce ATP [18, 19]. Despite its highly reduced genome, TM7x encodes a complete ADS operon, composed of *arcA* (TM7x_03440), *arcB* (TM7x_03435), *arcC* (TM7x_03425), and *arcE* (TM7x_03430). We previously presented indirect evidence suggesting that TM7x-encoded ADS is functional and likely essential for arginine catabolism [10]. To directly test its role, we targeted *arcE* and *arcA*, the two key control points of the pathway—*arcE* encodes the arginine transporter for substrate uptake, and *arcA* encodes the first enzyme initiating the ADS. Targeted mutagenesis was performed using a recently established protocol [20] (described in the Materials and Methods). Each gene was individually replaced with a hygromycin B resistance cassette (Fig. S1). Due to its obligate symbiotic lifestyle, each TM7x mutant was selected using hygromycin B and maintained in coculture with the host bacterium XH001. TM7x mutants were then isolated from coculture using an established method [9, 10] and confirmed by whole-genome sequencing.

**Figure 1:**
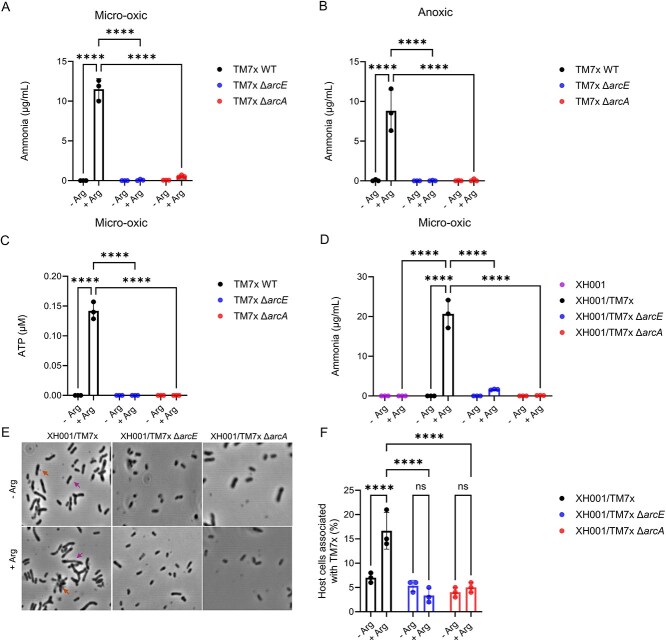
Evaluating the effect of *arc* gene deletions on ADS functionality in both free-living and host-associated conditions. (A) Ammonia production by TM7x WT, ∆*arcE*, and ∆*arcA* mutants cultured in RPMI supplemented with arginine under micro-oxic conditions. (B) Ammonia production by the same strains under anoxic conditions. (C) Intracellular ATP levels were measured under the same conditions as in panel A. (D) Ammonia levels in cocultures of TM7x and XH001, including host-only (XH001) and mutant combinations, demonstrating the functional contribution of ADS in symbiotic association. (E) Representative images showing host attachment efficiency of TM7x WT, TM7x Δ*arcE*, and TM7x Δ*arcA* strains under arginine-supplemented (+Arg) and arginine-free (–Arg) RPMI conditions. Orange arrows indicate TM7x cells, and purple arrows indicate host XH001 cells. (F) Quantification of host attachment percentages under each condition. Data are presented as mean ± standard error (n = 3). Statistical analysis was performed using two-way ANOVA with Tukey’s multiple comparisons test. *p* ≤ .05 (^*^), *p* ≤ .01 (^**^), *p* ≤ .001 (^***^), *p* ≤ .0001 (^****^).

We assessed ADS functionality of TM7x wildtype (WT), Δ*arcE*, and Δ*arcA* mutants by measuring ammonia production and intracellular ATP levels under free-living conditions. For TM7x WT and mutants, cells were isolated from respective co-cultures, and then incubated in SILAC RPMI 1640 Flex medium (lacking glucose and arginine) with or without arginine using an established protocol with minor modification [15, 19]. Since TM7x naturally inhabits the oral cavity, where it likely encounters both micro-oxic and anoxic niches, we measured ammonia production, a direct byproduct of arginine catabolism, under both conditions to capture its physiological range. We observed that in RPMI medium supplemented with arginine, TM7x WT produced significantly elevated levels of ammonia (~11.5 μg/ml) compared to the medium control under micro-oxic conditions (Fig. 1A), confirming active ADS function. Ammonia concentrations were comparable between micro-oxic (Fig. 1A) and anoxic conditions (Fig. 1B), indicating that TM7x WT utilizes arginine to a similar extent under both environments. Deletion of *arcE* resulted in almost no ammonia production (0.56 μg/ml) relative to WT (*P* < .0001), indicating that impaired arginine uptake limits ADS activity. Similarly, deletion of *arcA* resulted in complete loss of ammonia production, consistent with the loss of the key catabolic enzyme in the pathway (Fig. 1A and B).

We further quantified ATP levels to evaluate the impact of *arc* gene deletions on ATP production under micro-oxic condition. To reduce residual intracellular ATP carried over from their symbiotic growth with host bacteria, TM7x WT and mutants isolated from their respective co-cultures were first starved in RPMI medium for 24 hr and then re-inoculated into RPMI (with and without arginine) and incubated for another 24 hr. TM7x WT exhibited a significant increase in ATP levels in the presence of arginine compared to the no-arginine control, consistent with the activation of ADS pathway and ATP production. In contrast, both the Δ*arcE* and Δ*arcA* mutants showed no significant increase in ATP levels, confirming that both the transporter and enzyme are essential for ADS-mediated ATP generation (Fig. 1C).

Our results indicate that ArcE is essential in TM7x’s ability to utilize extracellular arginine when it is disassociated from host bacteria, consistent with its predicted role as an arginine–ornithine antiporter [10, 11]. To determine whether TM7x might acquire arginine through alternative mechanisms when forming symbiosis with its host, ammonia production in cocultures of XH001 with TM7x WT, Δ*arcE*, and Δ*arcA* mutants supplemented with arginine was monitored under micro-oxic condition. As XH001 lacks its own ADS [10], a control group using XH001 alone produced no detectable ammonia (Fig. 1D). In the host-associated state, TM7x WT produced robust levels of ammonia (~21 μg/ml), indicating active ADS function. In contrast, the Δ*arcE* mutant exhibited significantly reduced ammonia production (~1.6 μg/ml) even when forming episymbiosis with host bacteria, arguing against the presence of alternative mechanisms allowing for arginine acquisition directly from host bacteria. As expected, the Δ*arcA* mutant produced no detectable ammonia, confirming the essential role of arginine deiminase in ADS activity under host-associated conditions.

To further evaluate the temporal requirement for ArcE in arginine catabolism, we performed a time-course experiment with TM7x and TM7x Δ*arcE* cocultures incubated in RPMI medium with or without arginine. TM7x WT showed sustained and increasing ammonia production over 72 hr in arginine-supplemented RPMI, demonstrating efficient and prolonged arginine utilization (Fig. S2). In contrast, the Δ*arcE* mutant produced no detectable ammonia, confirming its inability to import and metabolize arginine without ArcE. Collectively, these findings confirm that the arginine/ornithine antiporter, ArcE is essential for ADS-dependent ammonia production and arginine uptake in both free-living and host-associated TM7x.

To determine whether ADS functionality provides a competitive advantage during host re-association, we performed a competition assay using mixed TM7x populations. We generated a TM7x derivative that expressed GFP (named TM7x-gfp), which displayed similar growth and symbiotic characteristics compared to TM7x WT. We then performed a competition assay where TM7x-gfp was mixed at a 1:1 ratio with either TM7x Δ*arcE* or Δ*arcA* mutant, and the mixed TM7x cells were used to infect XH001 using the established protocol [12]. Cocultures of XH001 and TM7x, following 5-day passages, were used to assess the competition outcome. The images acquired by phase-contrast (for transmission) and fluorescence microscopy were used to quantify TM7x-gfp versus Δ*arcE* or Δ*arcA* mutant abundance. In the TM7x-gfp + TM7x Δ*arcE* group, TM7x-gfp accounted for approximately 60% of the final population, whereas in the TM7x-gfp + TM7x Δ*arcA* group, TM7x-gfp representation increased to approximately 80% (Fig. S3). These results show that both *arcE* and *arcA* mutants are outcompeted by TM7x-gfp during host-associated growth, demonstrating that ADS-mediated arginine utilization confers a significant ecological and fitness advantage during re-association with XH001.

### Arginine metabolism supports efficient host attachment in TM7x

Our previous study showed that TM7x type IV pili mediated initial attachment to host bacteria is a critical step in establishing efficient episymbiosis and an energy-dependent process [20]. To test whether ATP from arginine catabolism supports this step, we performed host attachment of TM7x WT, Δ*arcE*, and Δ*arcA* mutant in RPMI with or without arginine. WT TM7x showed significantly higher attachment in arginine-supplemented media (~17% of host bound) than in arginine-free media (~7%) (*P* < .0001, Fig. 1E–F), suggesting that ATP generated from arginine catabolism via the ADS supports host attachment. In contrast, Δ*arcE* and Δ*arcA* mutants showed low attachment regardless of arginine availability, indicating that arginine metabolism via the ADS is required for efficient host binding under the condition tested.

### Arginine supplementation drives metabolic remodeling in isolated TM7x

**Figure 2:**
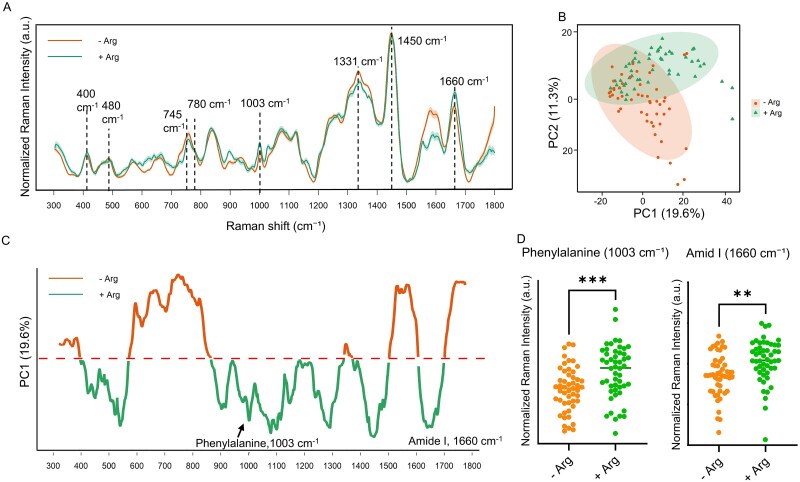
Raman spectroscopy and PCA analysis reveal arginine-dependent metabolic remodeling in isolated TM7x. (A) Raman spectra (mean ± standard error) of TM7x cultured in RPMI medium without arginine (orange) or with arginine (green) (n = 50 Raman spectra per condition), showing condition-specific spectral features. (B) PCA plot of Raman spectra demonstrating distinct clustering between two groups that indicates global metabolic shifts. Statistical analysis using PERMANOVA confirmed that the separation was significant (R^2^ = 0.08527, *p* = .001). (C) PC1 loadings highlighting Raman shifts that contribute most to the separation. (D) Quantitative analysis of integrated Raman intensities at key spectral markers: Phenylalanine (~1003 cm^−1^), and amide I (~1660 cm^−1^). Statistical comparisons were made using a two-tailed unpaired Student’s *t-*test. ^*^p ≤ .05, ^**^p ≤ .01.

To assess the metabolic impact of arginine on TM7x detached from the host bacteria, we performed Raman spectroscopy on TM7x WT cells incubated in RPMI with or without arginine under micro-oxic condition (Fig. 2A). Raman spectroscopy can probe the chemical composition of biomolecules in a label-free manner based on the vibrational information of chemical bonds [21, 22]. To visualize the overall biochemical divergence between the two conditions, we employed principal component analysis (PCA) to analyze the obtained Raman spectra (Fig. 2B), which revealed distinct clustering between the two conditions, with PC1 illustrating the major difference. Statistical analysis using PERMANOVA confirmed that the separation was significant (*R*^2^ = 0.09, F = 9.1, *P* = .001), indicating that arginine supplementation significantly altered the biochemical profile of TM7x.

We then examined specific spectral regions in detail (Fig. 2C, D, Supplemental Table 1). After PCA, PC1 (19.6% variance) revealed the Raman shifts that contributed most to the separation between +/− arginine conditions. The phenylalanine (1003 cm^−1^) and amide I (1660 cm^−1^) peaks [23–25] were the most prominent features, showing higher intensities in the arginine-supplemented condition. This increase suggests that arginine availability enhances protein content or alters protein conformation in TM7x, reflecting broader metabolic adjustments likely linked to ADS-mediated ATP generation.

### TM7x modulates host arginine metabolism via *arcA*-dependent signaling

To explore how TM7x may influence host bacterium arginine biosynthesis, we examined expression of arginine biosynthesis-related genes: *argR*, (APY09_07335) and *argG*, (APY09_07340) in XH001 monoculture and symbiotic co-cultures of XH001 with TM7x WT (XH001/TM7x WT) or Δ*arcA* mutant (XH001/TM7x Δ*arcA*). Comparative genomic analysis confirmed the absence of *argR* and *argG* homologs in the TM7x genome. We focused on Δ*arcA* since it directly disrupts ADS-mediated arginine catabolism. The XH001 *argR* and *argG* are predicted to be in the same operon. *argR* encodes a transcriptional regulator that represses arginine biosynthesis genes under high arginine conditions, while XH001 *argG* encodes argininosuccinate synthase which catalyzes the conversion of citrulline and aspartate into argininosuccinate. *argR* and *argG* were selected to represent both regulatory and structural genes involved in arginine biosynthesis in XH001.

**Figure 3:**
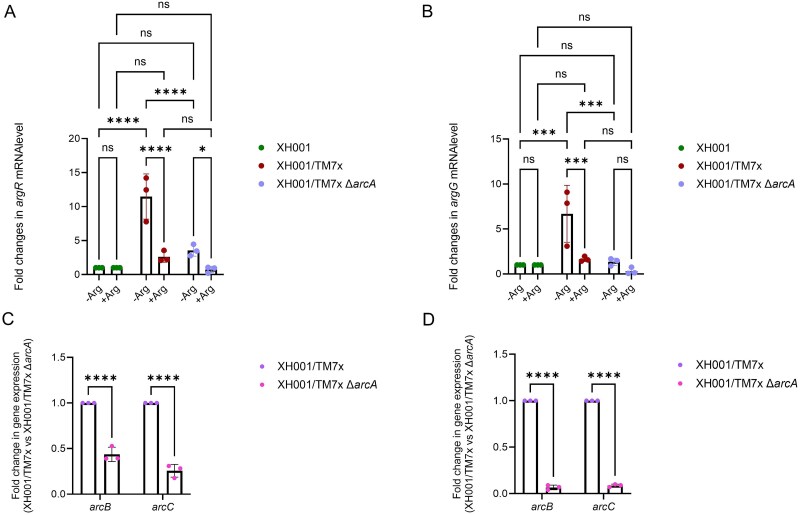
Crosstalk between XH001 and TM7x in arginine metabolism. (A–B) qPCR analysis of (A) *argR* and (B) *argG* expression in XH001 monoculture, XH001/TM7x, and XH001/TM7x ∆*arcA* coculture under arginine-deficient and arginine-supplemented conditions. (C–D) expression of *arcB* and *arcC* in WT and ∆*arcA* TM7x under (C) arginine-deficient and (D) arginine-supplemented conditions. Data are presented as mean ± standard error (n = 3). Statistical analysis was performed by two-way ANOVA with Tukey’s multiple comparison test. *p* ≤ .05 (^*^), *p* ≤ .01 (^**^), *p* ≤ .001 (^***^), *p* ≤ .0001 (^****^).

Under arginine-deficient conditions, the expression of *argR* and *argG* was significantly upregulated in the XH001/TM7x WT coculture compared to XH001 monoculture (Fig. 3A and B). Although genomic analysis indicates that XH001 is auxotrophic for de novo arginine biosynthesis, it retains partial functionality in the pathway, such as the ability to convert citrulline to arginine via *argG*. This upregulation may reflect XH001’s attempt to compensate for TM7x’s arginine demand, or alternatively, indicate metabolic competition for limited arginine precursors. In the XH001/TM7x Δ*arcA* coculture, both *argR* and *argG* were upregulated compared to monoculture, but their expression remained substantially lower than in XH001/TM7x WT coculture (Fig. 3A and B), indicating that a functional ADS is required for full activation of host arginine biosynthesis. Upon supplementation with exogenous arginine, expression of *argR* and *argG* was significantly reduced in both cocultures compared to arginine-depleted conditions, suggesting sufficient arginine for both organisms (Fig. 3A and B). Additionally, TM7x *arcB* and *arcC* expression were significantly lower in XH001/TM7x Δ*arcA* than in XH001/TM7x WT coculture, regardless of arginine availability, indicating that ArcA is crucial for proper expression of ADS genes (Fig. 3C and D). Comparative genomic analysis confirmed that XH001 lacks *arcB* and *arcC* homologs, ensuring that the expression measured for these genes derives solely from TM7x [10].

### Glucose metabolism in TM7x is enhanced under anoxic conditions via glycolytic and fermentative pathways

Although early genome annotations suggested that TM7x harbored only a partial glycolytic pathway [26], more recent genomic reanalysis reveals a broader capacity for glucose metabolism than initially believed [17]. Our genome survey identified most genes likely involved in glucose uptake and central carbon metabolism (Fig. S4), though key components such as phosphofructokinase (*pfk*) and glucose transporters were either missing or ambiguously annotated, leaving the mechanism of glucose uptake and utilization unresolved. TM7x_02415, originally annotated as a ribokinase, is located in the same operon as fructose-bisphosphate aldolase (TM7x_02420), and is predicted to function as a *pfkB*-type phosphofructokinase. This suggests that *pfk* is present but misannotated, with TM7x_02415 serving as a functional replacement of the canonical enzyme and a useful marker of glycolytic activity.

**Figure 4:**
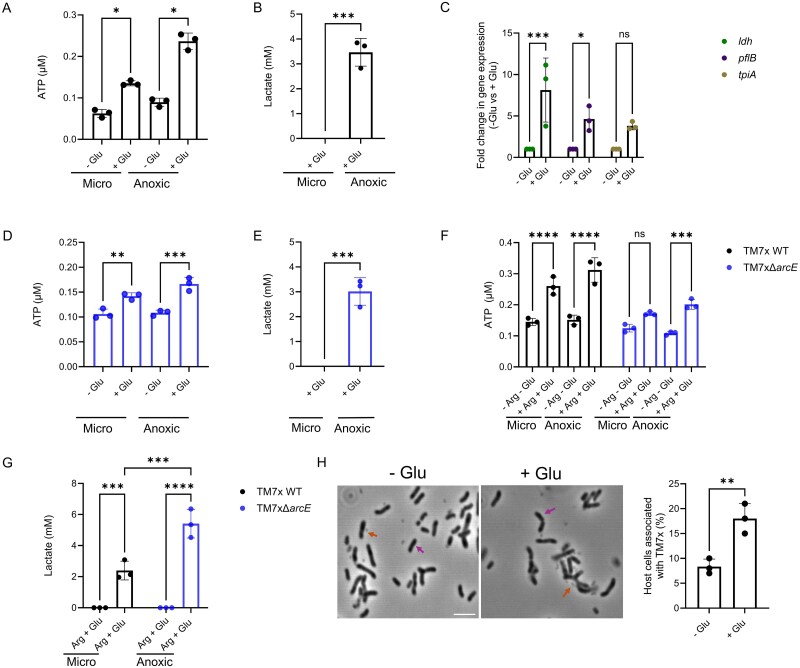
TM7x utilizes glucose via glycolysis and thereby promotes ATP production and fermentation, especially under anoxic conditions. (A) Glucose significantly increased ATP levels in TM7x WT under both micro-oxic (micro) and anoxic conditions. (B) Lactate production occurred only under anoxic conditions, indicating fermentation. (C) qPCR showed upregulation of glycolysis and fermentation genes (*ldh, tpiA*, and *pfk*) in glucose-supplemented cultures. (D–E) Similar ATP and lactate patterns were observed in the Δ*arcE* mutant. (F–G) Combined glucose and arginine supplementation revealed higher ATP production in WT and increased lactate in Δ*arcE*, suggesting ADS–glycolysis crosstalk. (H) TM7x WT showed increased host attachment in glucose-supplemented medium. Orange arrows indicate TM7x cells, and purple arrows indicate host XH001 cells. Data represent mean ± standard error from at least three biological replicates. Scale bar = 5 μm. Statistical analyses were performed using one/two-way ANOVA with unpaired *t*-test/ Tukey’s test. *p* ≤ 0.05 (^*^), *p* ≤ 0.01 (^**^), *p* ≤ 0.001 (^***^), *p* ≤ 0.0001 (^****^), ns = non-significant.

To test this predicted glycolytic capacity in TM7x, we measured ATP production under micro-oxic and anoxic conditions with glucose supplementation. We tested both micro-oxic and anoxic conditions because TM7x encounters varying oxygen levels in its environment, which may affect glucose metabolism and ATP production. The result showed that, while glucose supplementation led to a significant increase in intracellular ATP levels compared to RPMI alone under both conditions, TM7x generated significantly more ATP under anoxic than micro-oxic condition (Fig. 4A), indicating more active glucose utilization under anoxic condition. To confirm glucose metabolism, we measured lactate production as a marker of fermentation. We observed detectable lactate only under anoxic conditions in glucose-supplemented cultures (Fig. 4B), supporting the idea that TM7x engages in fermentation in the absence of oxygen. Under micro-oxic conditions, however, lactate was not detected, implying that pyruvate generated via glycolysis may be metabolized through alternative pathways, possibly involving mixed acid fermentation or other yet-to-be-identified routes to maintain redox balance. To confirm glucose utilization at the molecular level, we performed qPCR targeting key glycolytic and fermentation genes, including *ldh* (lactate dehydrogenase, TM7x_01995), which catalyzes the conversion of pyruvate to lactate during fermentation; *tpiA* (triosephosphate isomerase, TM7x_02890), involved in the interconversion of glycolytic intermediates and *pfk* (putative phosphofructokinase, TM7x_02415), a rate-limiting enzyme in glycolysis. Both *ldh* and *pfk* showed significantly elevated expression in glucose-supplemented conditions compared to RPMI alone, whereas *tpiA* also displayed increased expression in the presence of glucose, albeit not statistically significant (Fig. 4C). These results indicate that TM7x upregulates glycolytic and fermentative pathways to generate ATP in anoxic environments when glucose is available. While we have not yet generated deletions for these genes, future studies will focus on targeted gene knockouts to directly assess their functional roles in TM7x glucose metabolism.

Given that both the ADS and glycolysis generate ATP in TM7x, and prior studies have suggested potential metabolic crosstalk between them in other bacteria [27], we examined their potential cooperative effects in TM7x. We first examined ATP and lactate production in the Δ*arcE* mutant when provided with and without glucose. Similar to what was observed in the WT (Fig. 4A and B), glucose supplementation increased ATP levels in TM7x Δ*arcE* under both conditions (Fig. 4D) while lactate was produced only anoxically (Fig. 4E), confirming that TM7x Δ*arcE* mutant retains glycolytic function.

We assessed the effects of combined glucose and arginine to investigate the possible synergy between ADS and glycolysis. While both strains showed increased ATP with glucose alone (Fig. 4A and D), WT produced more ATP than Δ*arcE* when both substrates were present, suggesting ArcE-dependent ADS enhances energy yield (Fig. 4F). Interestingly, lactate production was higher in the Δ*arcE* mutant compared to WT under these conditions (Fig. 4G), possibly indicating a compensatory shift toward fermentation in the absence of ADS activity. These findings imply that ADS and glycolysis may act cooperatively in TM7x to optimize ATP production under nutrient-rich conditions.

To investigate the impact of glycolysis-mediated ATP production on the initial binding of TM7x to the host bacterium, we measured TM7x attachment efficiency in minimal media, using glucose-supplemented and glucose-free RPMI. These conditions were designed to test energization rather than reflect the natural physiological state. TM7x WT showed significantly higher attachment in the presence of glucose (~18% vs. ~8%, *P* < .0001; Fig. 4H), suggesting that glucose promotes host interaction, potentially by fueling energy-dependent attachment processes. The significant increase in TM7x attachment with glucose is comparable to the enhancement observed with arginine (Fig. 1F), indicating that both nutrients similarly promote TM7x viability and host association. While the mechanism remains unclear, this observation demonstrates that host association is an active process that depends on energization and suggests a metabolic link between glucose utilization and host engagement, possibly involving coordination with other ATP-generating pathways such as ADS.

### Raman spectroscopy reveals glucose and oxygen-dependent metabolic remodeling in TM7x

**Figure 5:**
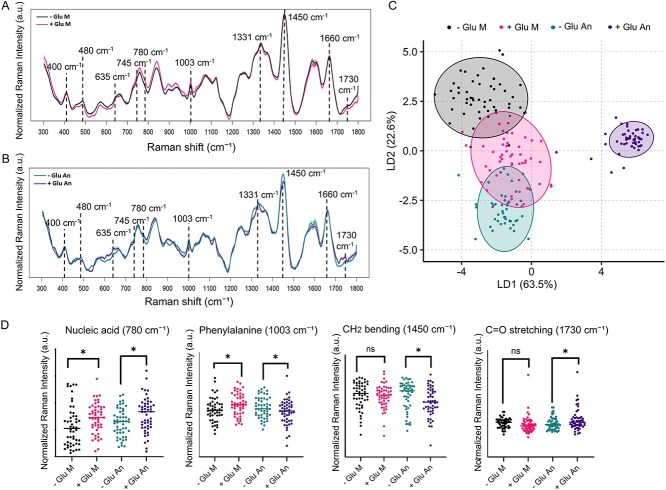
Raman spectroscopy and LDA revealed glucose-dependent metabolic remodeling in TM7x. (A, B) Averaged and normalized Raman spectra of isolated TM7x grown in RPMI alone (−Glu) versus RPMI supplemented with glucose (+Glu) under (A) micro-oxic (M) and (B) anoxic (An) conditions (n = 50 Raman spectra per condition). Each point represents an individual Raman spectrum; shaded areas indicate the standard error of the mean. (C) LDA plot demonstrates separation of Raman spectral profiles across four conditions. LD1 and LD2 capture the major variance in the dataset, illustrating the influence of glucose and redox environment on TM7x metabolic states. (D) Quantitative comparison of Raman band intensities at key metabolic marker wavelengths. Statistical significance was assessed by one-way ANOVA with Tukey’s multiple comparisons test. Significance levels are indicated as follows: *p* ≤ .05 (^*^), *p* ≤ .01 (^**^), *p* ≤ .001 (^***^), *p* ≤ .0001 (^****^), ns = non-significant.

To determine how glucose influences TM7x metabolism under different redox environments, we performed Raman spectroscopy in the range from 300 cm^−1^ to 1800 cm^−1^ on TM7x cells incubated with and without glucose, under both micro-oxic and anoxic conditions (Fig. 5A and B). Spectra were used to assess biochemical changes associated with glucose availability and oxygen levels. Linear Discriminant Analysis (LDA) of Raman spectra (Fig. 5C) showed clustering according to treatment conditions, with the first two linear discriminants (LD1 and LD2) explaining 63.47% and 22.57% of the variance, respectively. Pairwise PERMANOVA confirmed significant differences between all groups (*P* ≤ 0.001), with a large shift (R² ≈ 0.90) between groups under anoxic conditions, and smaller shift (*R*^2^ ≈ 0.58) between groups under micro-oxic conditions, indicating that both oxygen level and glucose availability strongly influence TM7x metabolic profiles.

Raman spectroscopy revealed distinct glucose-dependent metabolic responses in TM7x under micro-oxic versus anoxic conditions (Fig. 5D). The most likely Raman peak assignments and the corresponding glucose-dependent spectral changes observed under micro-oxic and anoxic conditions are summarized in Supplemental Table 2. Under micro-oxic growth with glucose, the 780 cm^−1^ (nucleic acids) [28] and 1003 cm^−1^ (phenylalanine) peaks [23–25] increased relative to the glucose-deficient control, indicating enhanced nucleic acid–associated signals and elevated aromatic amino acid content. In contrast, peaks associated with tyrosine (635 cm^−1^) [29], lipid/cell wall CH₂ bending modes (1450 cm^−1^) [23–25], and amide I/protein structure (1660 cm^−1^) [23–25] remained stable, suggesting that micro-oxic glucose supplementation does not substantially alter TM7x lipids or overall protein structural content. The 1730 cm^−1^ ester-linked lipid peak [30] also remained unchanged, indicating no measurable accumulation of esterified or storage lipids in micro-oxic conditions.

Under anoxic conditions with glucose, TM7x exhibited a distinct metabolic profile. The 780 cm^−1^ (nucleic acids) peak increased, indicating enhanced nucleic acid content, while the 1003 cm^−1^ (phenylalanine) peak decreased, reflecting reduced aromatic amino acid content under anaerobiosis. The 635 cm^−1^ (tyrosine) peak increased, indicating an anoxic-specific rise in tyrosine levels. The 1450 cm^−1^ CH₂ bending band decreased relative to the glucose-deficient control, reflecting reduced CH₂ content from lipids or cell wall components, or altered lipid packing. In contrast, the 1730 cm^−1^ lipid ester band increased specifically under anoxic conditions, suggesting selective enrichment of esterified or storage lipids. Peaks associated with 1606 cm^−1^ (tyrosine/phenylalanine) and 1660 cm^−1^ (amide I) remained stable, indicating minimal changes in overall protein structural content.

Overall, our results indicate that glucose consistently enhances nucleic acid-associated signals in TM7x across both oxygen environments, while lipid- and amino acid–associated responses diverge sharply. This pattern likely reflects oxygen-dependent lipid remodeling and selective amino acid modulation rather than uniform biosynthetic changes, suggesting a strategy for TM7x to adjust membrane composition, accumulate storage lipids, and maintain metabolic flexibility under anoxic conditions while remaining biosynthetically limited without its host.

To directly assess glucose uptake, we incubated TM7x cells with deuterated glucose (glucose-d_7_) and used Raman spectroscopy in the 600–3300 cm^−1^ range to detect the C–D bonds within the resultant biomolecules. This technique leverages the unique vibrational signature of C–D bonds, which appear in mostly Raman-silent region (1800–2800 cm^−1^) free from interference by other biomolecules, allowing sensitive and specific detection of deuterium incorporation into cellular metabolites [30–32]. Pure glucose-d_7_ solution was included as a positive control for C–D peak assignment (Fig. S5A). Raman spectra acquired from TM7x incubated with deuterated glucose under both micro-oxic and anoxic conditions exhibited a prominent peak at 2150 cm^−1^, consistent with the C–D stretching vibration of deuterium-labeled biomolecules (Fig. S5B). LDA plots further distinguished the deuterated group from the non-deuterated control one (Fig. S5C). Statistical analysis using PERMANOVA confirmed that the separation between the three conditions was significant (*R*^2^ = 0.47, *P* = .001). Quantification of the C–D to total (C–D + C–H) signal ratio (Fig. S5D) revealed significantly higher deuterium incorporation in deuterated samples compared to non-deuterated controls. This indicates that TM7x actively imports or metabolizes glucose regardless of oxygen availability. These results demonstrate that TM7x can utilize glucose, as evidenced by deuterium incorporation into cellular biomolecules, highlighting its metabolic flexibility across diverse environments.

### ADS and glycolysis pathways promote TM7x survival in free-floating phase

**Figure 6:**
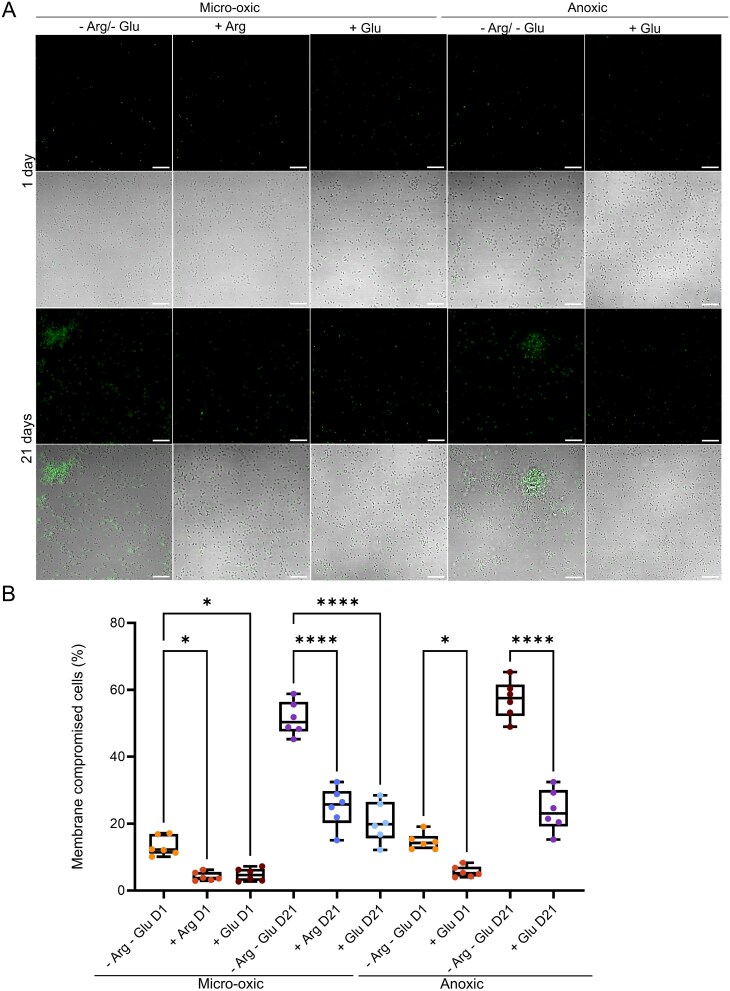
Arginine catabolism and glucose metabolism promote TM7x survival in the free-floating phase. (A) TM7x cells were isolated and incubated for 1 or 21 days in RPMI supplemented with or without arginine (Arg) or glucose (Glu) under micro-oxic and anoxic conditions. Cells were stained with SYTOX green to assess the membrane integrity. Representative images show the SYTOX fluorescence signal and merged fluorescence/transmission (FiJi adjusted) channels. Images were taken from at least six fields of view for each condition. (B) Cell membrane integrity was assessed by calculating the percentage of SYTOX green–positive cells relative to the total number of cells present. Data are presented as mean ± standard error (n = 6). Statistical significance was determined using one-way ANOVA. *p* ≤ .05 (^*^), *p* ≤ .01 (^**^), *p* ≤ .001 (^***^), *p* ≤ .0001 (^****^), ns = non-significant.

To determine whether ADS and glycolysis pathways influence TM7x viability in the absence of its host, we performed SYTOX Green assays over 21-days period under both micro-oxic and anoxic conditions, starting with TM7x cells from the same preparation. SYTOX Green penetrates cells with compromised membranes, thereby indicating the viability of bacteria. Under micro-oxic conditions, there were fewer SYTOX Green-positive (dead) cells in arginine or glucose-supplemented RPMI compared to RPMI alone sample at both day 1 and day 21 (Fig. 6A). A similar trend was observed under anoxic conditions, where glucose supplementation improved TM7x survival compared to RPMI alone (Fig. 6A). Quantitative analysis confirmed that both arginine and glucose significantly enhanced TM7x viability under nutrient-limited, free-floating conditions (Fig. 6B). These findings are consistent with those of [10], who showed that arginine helps maintain TM7x viability. Together, these results indicate that both the ADS and glycolytic pathways contribute to TM7x survival during horizontal transmission.

## Discussions

The lifestyle of *Saccharibacteria* members, particularly the human-associated strain TM7x, exemplifies extreme host bacteria dependency, genome reduction, and metabolic specialization. TM7x is an obligate episymbiont that requires an actinomycete host bacterium for sustainable growth and propagation. In particular, our study reveals that TM7x remains metabolically active and viable during transient free-floating phases by engaging specific energy-conservation pathways, the ADS and glycolysis.

Previous work highlighted the importance of ADS as a conserved feature among host-associated *Saccharibacteria*—including those in families such as Nanosynbacteraceae (G1), Saccharimonadaceae (G1, mammalian), Nanosyncoccaceae (G3), and Nanogingivalaceae (G6)—but lacked direct genetic validation due to technical limitations [10]. By generating *arcA* and *arcE* mutants using recently developed genetic tools [20, 33], our study now provides critical functional evidence, confirming that ADS plays a key role in TM7x energy maintenance. High-throughput Tn-seq screens previously identified that ADS genes are essential in a related *Saccharibacteria* species, *N. lyticus* strain ML1 (another oral G1 *Saccharibacteria*) [33]. Our findings clarify that *arcA* and *arcE* are not universally essential within *Saccharibacteria* but do play vital roles in the *Saccharibacteria* lifecycle, such as supporting enhanced fitness and enabling host interaction. The ADS system appears to be restricted to host-associated *Saccharibacteria*. In addition to providing ATP, the ADS system may protect, by generating ammonia, TM7x and its host bacterium from acid stress, a condition that is frequently encountered within the human oral cavity due to bacterial metabolism of dietary carbohydrates [10]. The lack of identifiable ADS or urease genes in environmental members of the phylum suggests they may rely on alternative mechanisms for intracellular pH regulation. Preliminary genomic surveys also indicate an absence of other ammonia-producing amino acid catabolic pathways, such as those involving glutamate, threonine, or serine degradation [34]. It is therefore possible that environmental *Saccharibacteria* employ alternative proton-regulating mechanisms, such as ATPases functioning as proton pumps, to maintain cytoplasmic homeostasis under fluctuating environmental conditions.

*Saccharibacteria* were originally named for their predicted ability to utilize sugars (Latin *saccharum*) [35], yet bioinformatic analyses have shown that many lineages lack complete glycolytic pathways [11, 16, 17]. Due to their obligate episymbiotic lifestyle and cultivation challenges, no direct experimental evidence had confirmed carbohydrate metabolism in *Saccharibacteria* until now. TM7x can utilize imported glucose for ATP generation through glycolysis and fermentation or store and accumulate glucose within glycogen polysaccharides. These findings expand the metabolic repertoire of *Saccharibacteria* beyond amino acid catabolism.

The coexistence of ADS and glycolysis in TM7x offers several advantages. Firstly, the ability to harness multiple nutrient sources diversifies TM7x’s ecological niches and supports survival in nutrient-variable microenvironments. Secondly, ADS remains active under both anoxic and micro-oxic conditions, aligning with the fluctuating oxygen levels in the oral cavity where TM7x can be found on the tongue dorsum (range of oxygen) or deep gingival pockets (no oxygen) [8]. Thirdly, glycolysis serves as an alternative ATP-generating mechanism, offering metabolic redundancy that supports TM7x during its free-floating transmission phase and ensures the energy reserves necessary for reinfection. Finally, given the need to toggle between nutrient sources depending upon environmental context, TM7x may deploy regulatory mechanisms to prioritize between ADS and glycolysis, a potential switch yet to be discovered.

A compelling aspect of our findings is the metabolic crosstalk between TM7x and its host. Under arginine-limited conditions, XH001 upregulates arginine biosynthesis-related genes (e.g., *argR* and *argG*) in response to TM7x symbiosis. As the host is likely an arginine auxotroph [11] this may be a result of competition or may be induced by a signaling mechanism from TM7x. Arginine metabolism also underlies TM7x’s ability to effectively attach to its host. ADS-derived ATP likely powers type IV pili twitching activity [19, 36] and the impaired attachment seen in *arcE* and *arcA* mutants confirms ADS’s role in providing energy for host interaction [10]. Beyond supporting its own survival, TM7x’s consumption of extracellular arginine reshapes host physiology, as reflected in the broad upregulation of host amino acid biosynthesis genes in coculture [11]. Given arginine’s central role in shaping oral biofilm ecology [37, 38], TM7x consumption of this resource could shift community structure beyond its immediate host, a possibility worth testing in future studies.

**Figure 7:**
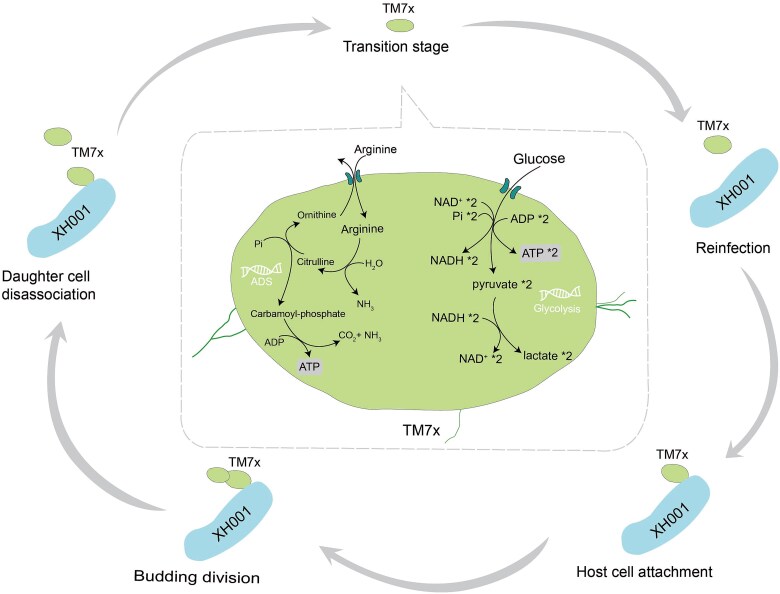
TM7x life-cycle and metabolic adaptation. The TM7x life-cycle involves a dynamic transition between host-associated and free-floating states, supported by distinct metabolic strategies. In the free-living state, TM7x cells remain viable by utilizing the arginine deiminase system (ADS) and glycolysis for ATP production. These energy-conservation pathways enable TM7x to survive independently and migrate toward a suitable host. Upon encountering a compatible host, such as *Schaalia odontolytica* (XH001), TM7x initiates infection by stably attaching to the host surface. Following attachment, TM7x undergoes asymmetric division through a budding process, producing distinct mother and daughter cells. The daughter cells then dissociate from the host, returning to the free-living state to complete the cycle.

Together, our findings support a refined five-stage life cycle model for TM7x (Fig. 7). Following asymmetric division on the host surface, daughter cells dissociate and enter a free-living phase during which ADS and/or glycolysis sustain ATP levels and viability. These pathways enable TM7x to persist in the environment long enough to locate and reinfect a new host cell, a process that may proceed through an initial tethering step mediated by type IV pili [20], followed by close-range attachment via cell wall–associated adhesins. This life cycle model highlights how organisms with highly reduced genomes coordinate metabolic processes to support survival and enable horizontal transmission between hosts.

In conclusion, this study establishes the functional role of the ADS in TM7x and provides the first experimental demonstration of glucose utilization in a *Saccharibacterium*. The presence of dual ATP-generating pathways enhances TM7x’s ability to persist in free-floating phase and reinitiate symbiosis, revealing a survival strategy that is both flexible and host-responsive. These insights lay the foundation for future investigations into gene function, host manipulation, and energy optimization in reduced-genome symbionts inhabiting complex microbial ecosystems.

## Materials and methods

### Bacterial strains and growth conditions

The TM7x strain (*N. lyticus* type strain, TM7x HMT-952) was originally co-isolated with its host bacterium, *S. odontolytica* (formerly Actinomyces odontolyticus) subspecies *actinosynbacter* strain XH001 (hereafter XH001), from the human oral cavity, and culture containing both referred to as co-culture (or XH001/TM7x). Wildtype or mutant co-cultures were cultivated in Brain Heart Infusion (BHI; BD Biosciences) medium, supplemented with or without 5% defibrinated sheep blood at 37°C under micro-oxic conditions (2% O₂, 5% CO₂, 93% N₂; Whitley A35, Don Whitley Scientific) [20]. For experiments requiring anoxic conditions, including TM7x transformations, cultures were grown inside an anaerobic chamber (Coy Laboratories, Grass Lake, MI, USA) supplied with a gas mixture of 5% hydrogen, 10% carbon dioxide, and 85% nitrogen.

### Construction and validation of TM7x knockout mutants

Knockout strains of TM7x were engineered using a hygromycin B resistance cassette system adapted from established protocols [20, 33]. To generate the constructs, 500 bp sequences flanking the target gene regions were amplified and assembled on either side of a codon-optimized hygromycin resistance gene. This gene was placed under the control of the *tuf* promoter from *Southlakia epibionticum*, and the full cassette was assembled using NEBuilder HiFi DNA Assembly (New England Biolabs, Ipswich, MA, USA). TM7x transformation was carried out in coculture with XH001 under strictly anoxic conditions using 1 μg of the linear DNA in a total volume of 500 μL. After 6-hr, 3 mL of BHI medium containing diluted XH001 (OD_600_ = 0.05) and hygromycin B (final concentration 150 μg/mL) was added. Cultures were passaged anoxically four times, then grown under micro-oxic conditions without antibiotics. Successful transformants were identified by a 70% enrichment in TM7x abundance between passages 4 and 6. Colonies exhibiting irregular morphology were identified using a Ken-A-Vision T-22021 stereomicroscope (Kansas City, MO, USA), based on previously established colony characteristics that indicate TM7x presence [39]. Candidate colonies were screened by PCR, and successful recombinants were verified by whole-genome sequencing (WGS) performed by Psomagen (Rockville, MD, USA). Primers used in mutant construction are listed in Supplemental Table 3.

### Generation of GFP-expressing *N. lyticus* TM7x

To generate fluorescent *N. lyticus*, linear constructs were designed to insert both a fluorescence cassette and a hygromycin B resistance cassette (Hph) into the previously published Neutral Site 1 (NS1) via homologous recombination [20, 33]. To navigate the underexplored complexity of *Saccharibacteria* gene expression a range of fluorescent proteins (sfGFP. mNeonGreen, and mCherry) and promoters from *N. lyticus* (pTuf, pRpsJ, and pArcA) were screened to identify the best candidates. Constructs containing sfGFP and mCherry were graciously provided by Drs. Joseph Mougous and Larry Galagher. For each construct, 300 bp to 500 bp homology arms from upstream and downstream of NS1 were amplified and assembled onto one fluorophore/promoter pair, and one Hph. Linear products were amplified and transformed into TM7x via the previously published protocol [20]. All constructs could be successfully integrated into the *N. lyticus* genome, as validated via Sanger sequencing of the insertion site, however mNeonGreen bearing strains were non-fluorescent and a subset of mCherry bearing populations were non-fluorescent. Optimal fluorescence was observed in *N. lyticus* NS1::pTuf-sfGFP/pRpsJ-Hph, henceforth *N. lyticus* TM7x-gfp, which was used for all the competition assay.

### Isolation of free-floating TM7x cells

TM7x cells were isolated from cocultures using previously described protocols to minimize host bacterial debris contamination [9, 10, 39, 40]. Briefly, ~400 mL XH001/TM7x coculture grown in BHI was transferred into 50 mL tubes, vortexed to dislodge loosely attached TM7x, and centrifuged at 3000 × g for 5 min to pellet host cells. Supernatants were filtered through 0.45 μm PVDF filters (Millipore Sigma) to remove residual host bacteria, then centrifuged at 20000 × g for 1 h at 4°C to pellet TM7x. Phase-contrast microscopy (Nikon Eclipse E400) was used to confirm the presence and morphology of isolated TM7x cells. For storage, purified TM7x preparations were adjusted to an OD_600_ of 0.2–0.4 and frozen at −80°C in phosphate-buffered saline (PBS) containing 15% glycerol to preserve cell viability.

### TM7x substrate utilization under defined conditions

Purified TM7x cells were washed twice with 1 mL of SILAC RPMI 1640 Flex medium (lacking glucose and phenol red; Thermo Fisher Scientific, Cat# A2494201) to remove residual BHI components. The washed cells were then resuspended in 5 mL of RPMI and incubated for 24-hr at 37°C in a micro-oxic chamber. TM7x cells were pelleted and resuspended in 12 mL of fresh RPMI medium either alone or supplemented with 10 mM L-arginine monohydrochloride and/or 10 mM D-glucose. The suspension was split into three tubes for equal cell input. Extracellular ammonia, intracellular ATP, and extracellular L-lactate was measured using the Ammonia Assay Kit (Sigma-Aldrich, Cat# AA0100), ATP Colorimetric/Fluorometric Assay Kit (Abcam, Cat# ab83355/K354), and L-Lactate Assay Kit (Abcam, Cat# ab65330), respectively, following each manufacturer’s instructions to evaluate TM7x metabolic activity.

### TM7x host attachment assay

Purified TM7x strains (WT and mutants) were normalized to an OD_600_ of 0.1 and introduced to XH001 at a multiplicity of infection (MOI) of 0.1. Co-cultures were incubated in RPMI medium, with or without supplementation of 10 mM L-arginine monohydrochloride and/or 10 mM D-glucose, at 37°C under micro-oxic conditions for 24 hr. The percentage of host cells associated with TM7x was quantified using phase-contrast microscopy (Nikon Eclipse E400), with 8–10 fields (~ 350 cells) manually counted per biological replicate. We define a “bound cell” as any host cell observed in direct contact with one or more episymbionts during imaging.

### Raman spectroscopy analysis

We used Raman microspectroscopy to examine the chemical composition of individual TM7x cells, following approaches commonly applied in microbiology [41]. Purified TM7x cells were cultured in RPMI medium supplemented with either 10 mM L-arginine monohydrochloride, 10 mM glucose or 10 mM deuterated D-glucose (glucose-d_7_) (Sigma Aldrich, 552 003-100MG) for 24 hr at 37°C. Cells were then fixed in 10% neutral buffered formalin (Sigma-Aldrich) for 60 minutes at room temperature and washed three times with sterile water and air-dried on aluminum-coated Raman substrates. Raman spectra were collected using an HR Evolution confocal Raman microscope (Horiba Jobin-Yvon, France) equipped with a 532 nm Nd:YAG laser. The laser output was attenuated to 8 mW at the sample using 25% neutral density filters. A 100× objective lens (NA = 0.6) was employed to focus the laser onto TM7x cell aggregates, producing a spot size of approximately 1 μm^2^. TM7x cells tend to form small aggregates, so the laser may occasionally capture signals from multiple cells, contributing to spectral variability. Scattered Raman signals were detected using a charge-coupled device (CCD) detector cooled to −70°C. Spectra were recorded in the range of 300–1800 cm^−1^ for non-deuterated samples and 600–3300 cm^−1^ for deuterated glucose, using a 600 grooves/mm diffraction grating, providing a spectral resolution of 1.5 cm^−1^. Acquisition settings included an integration time of 10 seconds with two accumulations per spectrum. Spectra were processed in LabSpec 6 (cosmic ray removal, baseline correction, vector normalization) and analyzed in RStudio (2024.09.1) using custom scripts (https://github.com/nusratnahar17/ADS-project-publication/blob/main/ADS_project.R).

### Viability assay of TM7x under nutrient supplementation

Isolated TM7x cells were washed twice to remove residual BHI. and divided equally per condition. Cells were resuspended in RPMI with and without 10 mM L-arginine monohydrochloride or 10 mM D-glucose at 37°C. At days 1 and day 21, cells were harvested and pelleted by centrifugation at 17 000 × g for 10 minutes at 4°C. Cells were stained with 5 μM SYTOX Green nucleic acid stain (Invitrogen, Cat# S7020), for 30 minutes in the dark, followed by two washes with sterile water to remove unbound dye. Cells were then fixed with 4% formaldehyde for 1 hr at room temperature. Imaging was performed using a Zeiss LSM 880 confocal laser scanning microscope with a 63× oil immersion objective (NA = 1.4). Approximately 250 cells from six different fields of view were analyzed per biological replicate, and three biological replicates per condition were included.

### Quantitative real-time PCR

Bacterial cultures were grown in BHI broth at 37°C until mid-log phase (OD_600_ ≈ 0.3–0.4). Total RNA was extracted from 2 mL of culture using the MasterPure Complete DNA and RNA Purification Kit (EPICENTRE, Cat# MC85200 and MC89010) following the manufacturer’s protocol. Complementary DNA (cDNA) was synthesized from the purified RNA using the HiFiScript cDNA Synthesis Kit (CoWin BioSciences, Cambridge, MA, USA) according to the manufacturer’s instructions. Quantitative real-time PCR (qRT-PCR) was performed in triplicate using 10 ng cDNA, 10 μM of each primer, and PowerUp™ SYBR™ Green Master Mix (Applied Biosystems). 16S rRNA was used as the internal control. Reactions were carried out on a QuantStudio 3 Real-Time PCR System (Thermo Fisher Scientific, Waltham, MA, USA) with the following cycling conditions: 95°C for 1 minute, followed by 40 cycles of 95°C for 30 seconds and 60°C for 30 seconds. Relative gene expression was calculated by 2^−ΔΔCT^. Primer details are in Supplementary Table 3.

### Statistical analysis

All statistical analyses were performed in Prism 10.3.1 (GraphPad Software). Depending on the dataset, comparisons were made using unpaired *t*-tests or one−/two-way ANOVA followed by Tukey’s multiple-comparison test. Statistical significance was defined as ^*^P < .05, ^**^P < .01, ^***^P < .001, ^****^P < .0001.

## Supplementary Material

wraf288_Supplementary_Figures_and_Tables_final

## Data Availability

All data supporting the findings of this study are included in the main text and Supplementary Materials. The raw Raman spectroscopy datasets generated in this study have been deposited in the MicrobioRaman database and are publicly available under the BioStudies accession numbers S-MBRS16 and S-MBRS17.

## References

[1] Moreira D, Zivanovic Y, López-Archilla AI. et al. Reductive evolution and unique predatory mode in the CPR bacterium *Vampirococcus lugosii*. *Nat Commun* 2021;12:2454. 10.1038/s41467-021-22762-4 33911080 PMC8080830

[2] Naud S, Ibrahim A, Valles C. et al. Candidate phyla radiation, an underappreciated division of the human microbiome, and its impact on health and disease. *Clin Microbiol Rev* 2022;35:e0014021. 10.1128/cmr.00140-21 35658516 PMC9491188

[3] Brown CT, Hug LA, Thomas BC. et al. Unusual biology across a group comprising more than 15% of domain bacteria. *Nat.* 2015;523:208–11. 10.1038/nature14486 26083755

[4] McLean JS, Bor B, Kerns KA. et al. Acquisition and adaptation of ultra-small parasitic reduced genome bacteria to mammalian hosts. *Cell Rep* 2020;32:107939. 10.1016/j.celrep.2020.107939 32698001 PMC7427843

[5] He X, McLean JS, Edlund A. et al. Cultivation of a human-associated TM7 phylotype reveals a reduced genome and epibiotic parasitic lifestyle. *Proc Natl Acad Sci USA* 2015;112:244–9. 10.1073/pnas.1419038112 25535390 PMC4291631

[6] Cross KL, Campbell JH, Balachandran M. et al. Targeted isolation and cultivation of uncultivated bacteria by reverse genomics. *Nat Biotechnol* 2019;37:1314–21. 10.1038/s41587-019-0260-6 31570900 PMC6858544

[7] Bor B, Bedree JK, Shi W. et al. *Saccharibacteria* (TM7) in the human oral microbiome. *J Dent Res* 2019;98:500–9. 10.1177/0022034519831671 30894042 PMC6481004

[8] Baker JL, Mark Welch JL, Kauffman KM. et al. The oral microbiome: diversity, biogeography and human health. *Nat Rev Microbiol* 2024;22:89–104. 10.1038/s41579-023-00963-6 37700024 PMC11084736

[9] Bor B, Poweleit N, Bois JS. et al. Phenotypic and physiological characterization of the epibiotic interaction between TM7x and its basibiont *Actinomyces*. *Microb Ecol* 2016;71:243–55. 10.1007/s00248-015-0711-7 26597961 PMC4688200

[10] Tian J, Utter DR, Cen L. et al. Acquisition of the arginine deiminase system benefits epiparasitic *Saccharibacteria* and their host bacteria in a mammalian niche environment. *Proc Natl Acad Sci USA* 2022;119:e2114909119. 10.1073/pnas.2114909119 PMC8764695 34992141

[11] Hendrickson EL, Bor B, Kerns KA. et al. Ultrasmall epibiont *Nanosynbacter lyticus* strain TM7x and host bacteria transcriptional activity after initial host parasitism. *J Oral Microbiol* 2024;16:2287349. 10.1080/20002297.2023.2287349 38188073 PMC10768705

[12] Utter DR, He X, Cavanaugh CM. et al. The saccharibacterium TM7x elicits differential responses across its host range. *ISME J* 2020;14:3054–67. 10.1038/s41396-020-00736-6 32839546 PMC7784981

[13] Dong Y, Chen YY, Snyder JA. et al. Isolation and molecular analysis of the gene cluster for the arginine deiminase system from *Streptococcus gordonii* DL1. *Appl Environ Microbiol* 2002;68:5549–53. 10.1128/aem.68.11.5549-5553.2002 12406748 PMC129940

[14] Cunin R, Glansdorff N, Piérard A. et al. Biosynthesis and metabolism of arginine in bacteria. *Microbiol Rev* 1986;50:314–52. 10.1128/mr.50.3.314-352.1986 3534538 PMC373073

[15] Xu B, Yang X, Zhang P. et al. The arginine deiminase system facilitates environmental adaptability of *Streptococcus equi* ssp. zooepidemicus through pH adjustment. *Res Microbiol* 2016;167:403–12. 10.1016/j.resmic.2016.03.005 27068185

[16] Srinivas P, Peterson SB, Gallagher LA. et al. Beyond genomics in *Patescibacteria*: a trove of unexplored biology packed into ultrasmall bacteria. *Proc Natl Acad Sci USA* 2024;121:e2419369121. 10.1073/pnas.2419369121 39665754 PMC11665869

[17] Figueroa-Gonzalez PA, Bornemann TLV, Adam PS. et al. *Saccharibacteria* as organic carbon sinks in hydrocarbon-fueled communities. *Front Microbiol* 2020;11:587782. 10.3389/fmicb.2020.587782 33424787 PMC7786006

[18] Van Wuyckhuyse BC, Perinpanayagam HE, Bevacqua D. et al. Association of free arginine and lysine concentrations in human parotid saliva with caries experience. *J Dent Res* 1995;74:686–90. 10.1177/00220345950740021001 7722066

[19] Wijeyeweera RL, Kleinberg I. Arginolytic and ureolytic activities of pure cultures of human oral bacteria and their effects on the pH response of salivary sediment and dental plaque in vitro. *Arch Oral Biol* 1989;34:43–53. 10.1016/0003-9969(89)90045-9 2675800

[20] Grossman AS, Lei L, Botting JM. et al. *Saccharibacteria* deploy two distinct type IV pili, driving episymbiosis, host competition, and twitching motility. *ISME J.* 2025;19:wraf119. 10.1093/ismejo/wraf119 PMC12206443 40488407

[21] Cheng JX, Xie XS. Vibrational spectroscopic imaging of living systems: an emerging platform for biology and medicine. *Sci.* 2015;350:aaa8870. 10.1126/science.aaa8870 26612955

[22] Dong P-T, Tian J, Kobayashi-Kirschvink KJ. et al. Episymbiotic *Saccharibacteria* induce intracellular lipid droplet production in their host bacteri. *ISME J* 2024;18:wrad034. 10.1093/ismejo/wrad034 PMC10939385 38366018

[23] Hsu C-C, Xu J, Brinkhof B. et al. A single-cell Raman-based platform to identify developmental stages of human pluripotent stem cell-derived neurons. *Proc Natl Acad Sci USA* 2020;117:18412–23. 10.1073/pnas.2001906117 32694205 PMC7414136

[24] Rivas-Arancibia S, Rodríguez-Martínez E, Badillo-Ramírez I. et al. Structural changes of amyloid beta in hippocampus of rats exposed to ozone: a Raman spectroscopy study. *Front Mol Neurosci* 2017;10:137. 10.3389/fnmol.2017.00137 28588448 PMC5438967

[25] Anastassopoulou J, Kyriakidou M, Mamareli V. et al. The influence of UV irradiation on diabetic mice skin: a vibrational FT-IR and Raman spectroscopic study. *Chromatogr Spectrosc Tech* 2019;2:21–27. 10.36959/326/768

[26] Hendrickson EL, Bor B, Kerns KA. et al. Transcriptome of epibiont *Saccharibacteria Nanosynbacter lyticus* strain TM7x during the establishment of symbiosis. *J Bacteriol* 2022;204:e0011222. 10.1128/jb.00112-22 35975994 PMC9487520

[27] Liu Y, Liu S, Zhi Q. et al. Arginine-induced metabolomic perturbation *in Streptococcus mutans*. *J Oral Microbiol* 2022;14:2015166. 10.1080/20002297.2021.2015166 35024088 PMC8745357

[28] Pezzotti G . Raman spectroscopy in cell biology and microbiology. *J Raman Spectrosc* 2021;52:2348–443. 10.1002/jrs.6204

[29] Baron VO, Chen M, Hammarstrom B. et al. Real-time monitoring of live mycobacteria with a microfluidic acoustic-Raman platform. *Commun Biol* 2020;3:236. 10.1038/s42003-020-0915-3 32409770 PMC7224385

[30] Lambert A, Bougrioua F, Abbas O. et al. Temperature dependent Raman and X-ray diffraction studies of anhydrous milk fat. *Food Chem* 2018;267:187–95. 10.1016/j.foodchem.2017.09.006 29934155

[31] Berry D, Mader E, Lee TK. et al. Tracking heavy water (D2O) incorporation for identifying and sorting active microbial cells. *Proc Natl Acad Sci USA* 2015;112:E194–203. 10.1073/pnas.1420406112 25550518 PMC4299247

[32] Yasuda M, Takeshita N, Shigeto S. Deuterium-labeled Raman tracking of glucose accumulation and protein metabolic dynamics in *Aspergillus nidulans* hyphal tips. *Sci Rep* 2021;11:1279. 10.1038/s41598-020-80270-9 33446770 PMC7809412

[33] Wang Y, Gallagher LA, Andrade PA. et al. Genetic manipulation of *Patescibacteria* provides mechanistic insights into microbial dark matter and the epibiotic lifestyle. *Cell.* 2023;186:4803–4817.e13. 10.1016/j.cell.2023.08.017 37683634 PMC10633639

[34] He W, Liang H, Li W. et al. Revealing an unprecedented diversity of episymbiotic *Saccharibacteria* in a high-quality genome collection. *npj Biofilms and Microbiomes* 2024;10:153. 10.1038/s41522-024-00617-2 39702451 PMC11659515

[35] Albertsen M, Hugenholtz P, Skarshewski A. et al. Genome sequences of rare, uncultured bacteria obtained by differential coverage binning of multiple metagenomes. *Nat Biotechnol* 2013;31:533–8. 10.1038/nbt.2579 23707974

[36] Geiger CJ, O'Toole GA. Evidence for the type IV pilus retraction motor *pilT* as a component of the surface sensing system in *Pseudomonas aeruginosa*. *J Bacteriol* 2023;205:e0017923. 10.1128/jb.00179-23 37382531 PMC10367593

[37] Nascimento MM, Alvarez AJ, Huang X. et al. Arginine metabolism in supragingival oral biofilms as a potential predictor of caries risk. *JDR Clin Trans Res* 2019;4:262–70. 10.1177/2380084419834234 31039043 PMC6572888

[38] Zheng X, He J, Wang L. et al. Ecological effect of arginine on oral microbiota. *Sci Rep* 2017;7:7206. 10.1038/s41598-017-07042-w 28775282 PMC5543048

[39] Bor B, McLean JS, Foster KR. et al. Rapid evolution of decreased host susceptibility drives a stable relationship between ultrasmall parasite TM7x and its bacterial host. *Proc Natl Acad Sci USA* 2018;115:12277–82. 10.1073/pnas.1810625115 30442671 PMC6275545

[40] Bor B, Collins AJ, Murugkar PP. et al. Insights obtained by culturing *Saccharibacteria* with their bacterial hosts. *J Dent Res* 2020;99:685–94. 10.1177/0022034520905792 32075512 PMC7243422

[41] Lee K, Landry Z, Pereira F. et al. Raman microspectroscopy for microbiology. *Nat Rev Methods Primers* 2021;1:80. 10.1038/s43586-021-00075-6

