# Supplementary material for: Ultrasmall episymbiont Nanosynbacter lyticus employs multiple ATP-generating metabolic pathways during horizontal transmission
Source: ISME J. 2025 Dec 29;20(1):wraf288. doi: 10.1093/ismejo/wraf288 (PMC12815258; doi:10.1093/ismejo/wraf288)
Supplement: wraf288_Supplementary_Figures_and_Tables_final [file wraf288_supplementary_figures_and_tables_final.docx]

**Ultrasmall episymbiont *Nanosynbacter* *lyticus* employs multiple ATP-generating metabolic pathways during horizontal transmission**

**Nusrat Nahar^1^, Pu-Ting Dong^1^, Jing Tian^2^, Alex S. Grossman^1^, Erik L. Hendrickson^3^, Kristopher A. Kerns^3^, Mary Ellen Davey^1^, Batbileg Bor^1^, Jeffrey S. McLean^3,4,5^, Xuesong He^1#^**

1. Department of Microbiology, ADA Forsyth Institute, Somerville MA, 02143, USA
2. Department of Pediatric Dentistry, Peking University School and Hospital of Stomatology, Beijing 100081, China
3. Department of Periodontics, University of Washington, Seattle WA, 98195, USA
4. Department of Microbiology, University of Washington, Seattle WA, 98109, USA
5. Department of Oral Health Sciences, University of Washington, Seattle WA, 98195, USA

# Corresponding author, [xhe@forsyth.org](mailto:xhe@forsyth.org)

Supplemental Figures and Tables


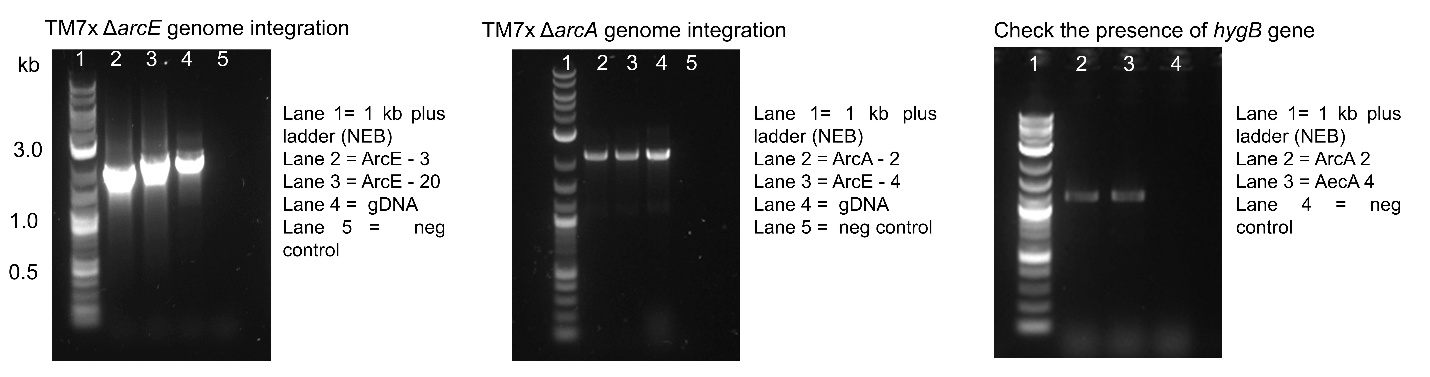


**Figure S1:** Knockout of *arcE* and *arcA* genes. Confirmation of *arcE* and *arcA* gene knockouts in TM7x. PCR verification of targeted deletions using gene-specific primers shows successful removal of the *arcE* and *arcA* coding sequences in the respective mutant strains with hygromycin B cassette. Marker sizes are indicated.


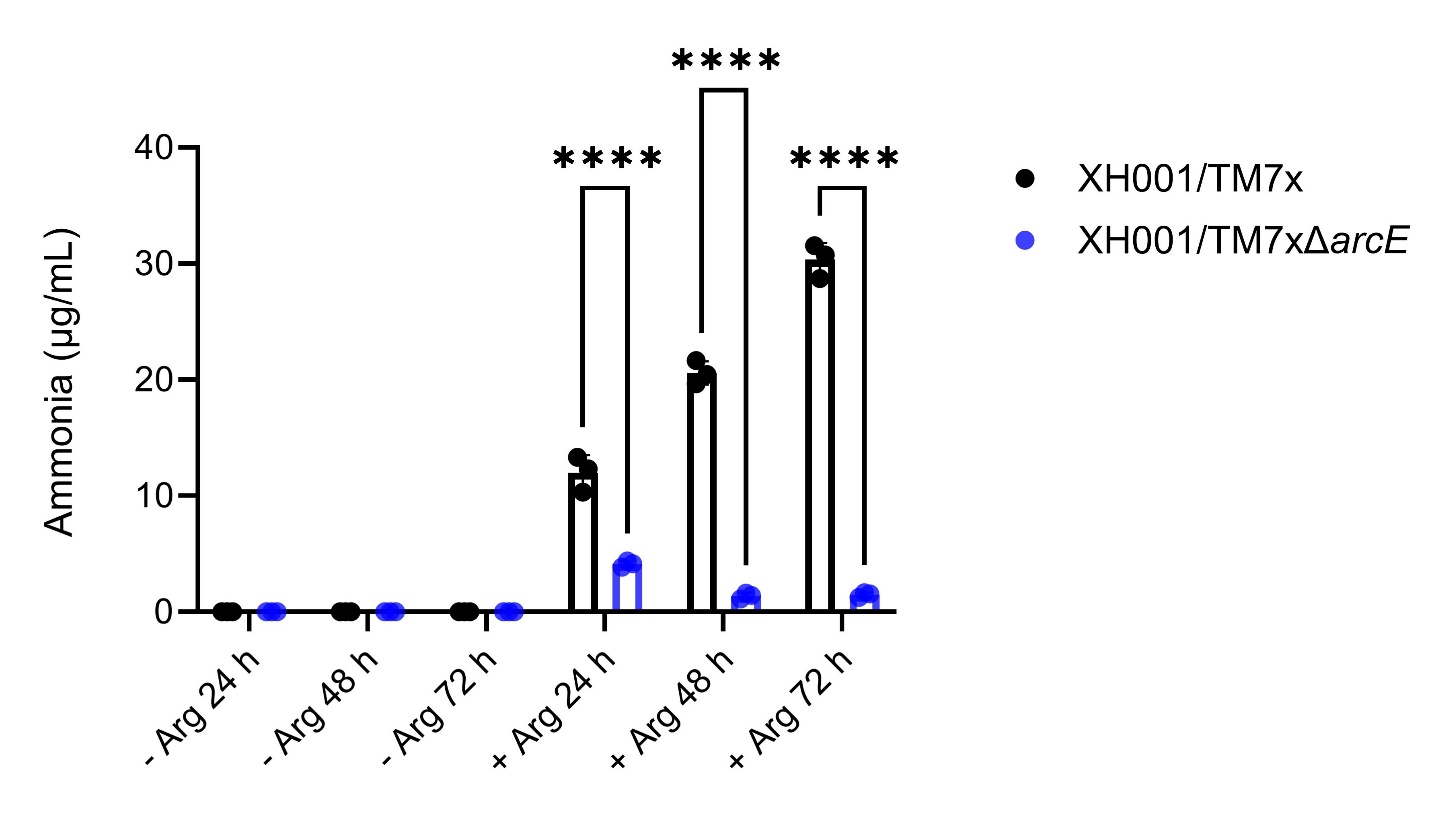


**Figure S2:** *arcE* is essential for arginine uptake. Cocultures of *S. odontolytica* XH001 with either wild-type (WT) or ∆*arcE* TM7x were incubated in RPMI medium supplemented with (+ Arg) or without (− Arg) 10 mM arginine. Ammonia levels were quantified at 0, 24, 48, and 72 hr. WT cocultures in +Arg conditions showed sustained and increasing ammonia production, consistent with active arginine catabolism via the ADS pathway. In contrast, ∆*arcE* cocultures exhibited minimal ammonia production under all conditions, confirming the critical role of ArcE in arginine uptake. Data represent mean ± SD from three biological replicates. Significance is indicated as *P* ≤ 0.05 (**), ≤ 0.01 (**), ≤ 0.001 (****), ≤ 0.0001 (****).


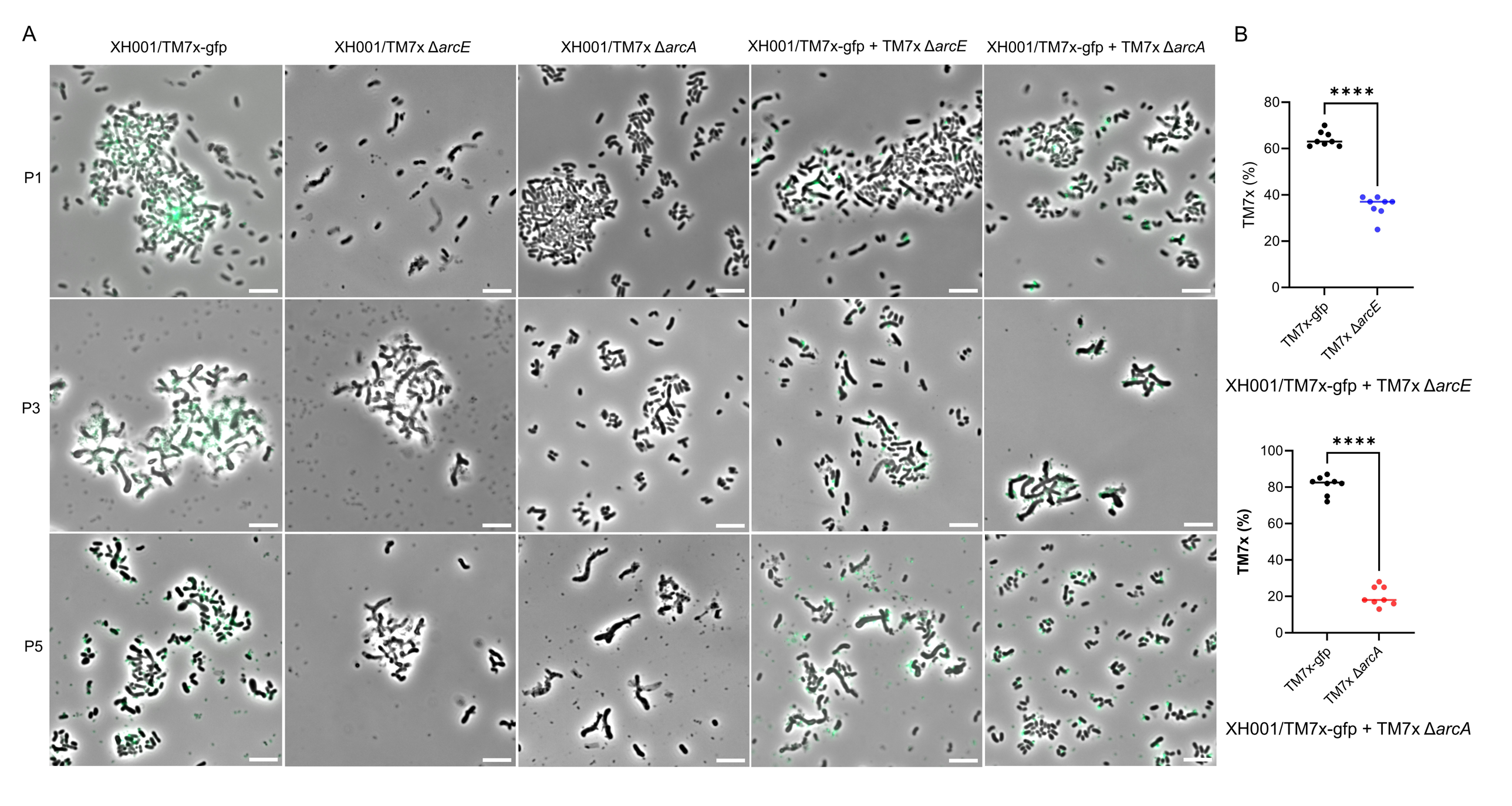


**Figure S3:** Competitive fitness of TM7x-gfp versus Δ*arcE* and Δ*arcA* mutants during host-associated growth. (A) Individual and mixed populations of GFP-labeled TM7x (named TM7x-gfp) and either Δ*arcE* or Δ*arcA* mutants were used to infect XH001. Cocultures were maintained over a five-day passage experiment; early passages were difficult to quantify due to TM7x clumping. Fluorescence microscopy images were used to determine the relative abundance of TM7x-gfp and mutant cells. (B) After passage 5, TM7x-gfp cells account for ~60% and ~80% of the total TM7x population in the two competition groups where TM7x-gfp cells compete with *arcE* and *arcA* mutants, respectively. Data are presented as mean ± standard error (n = 8). Statistical analysis was performed using unpaired Student’s t-test. Significance is indicated as *P* ≤ 0.05 (**), ≤ 0.01 (****), ≤ 0.001 (****), ≤ 0.0001 (****).


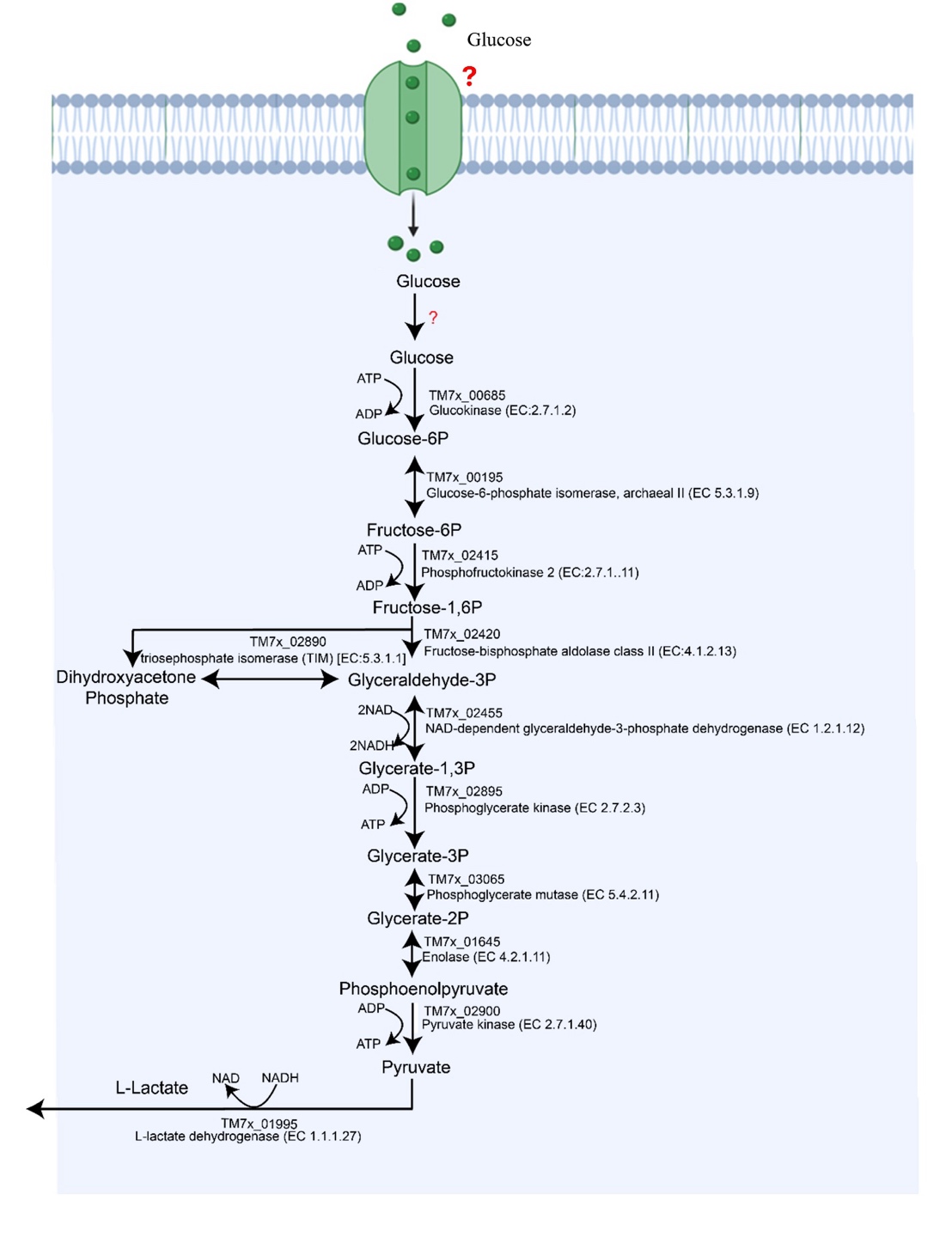


**Figure S4:** Predicted glycolytic pathway in *N. lyticus* TM7x based on genomic annotation. The schematic illustrates the core steps of glycolysis, presenting genes identified in the TM7x genome. The figure reflects TM7x’s capacity to metabolize glucose through a modified glycolytic pathway. Notably, the canonical phosphofructokinase (*pfk*) gene appears to be absent or misannotated. Instead, a ribokinase homolog (TM7x_02415) is predicted to catalyze the conversion of fructose-6-phosphate to fructose-1,6-bisphosphate, suggesting a potential non-canonical bypass at this step.


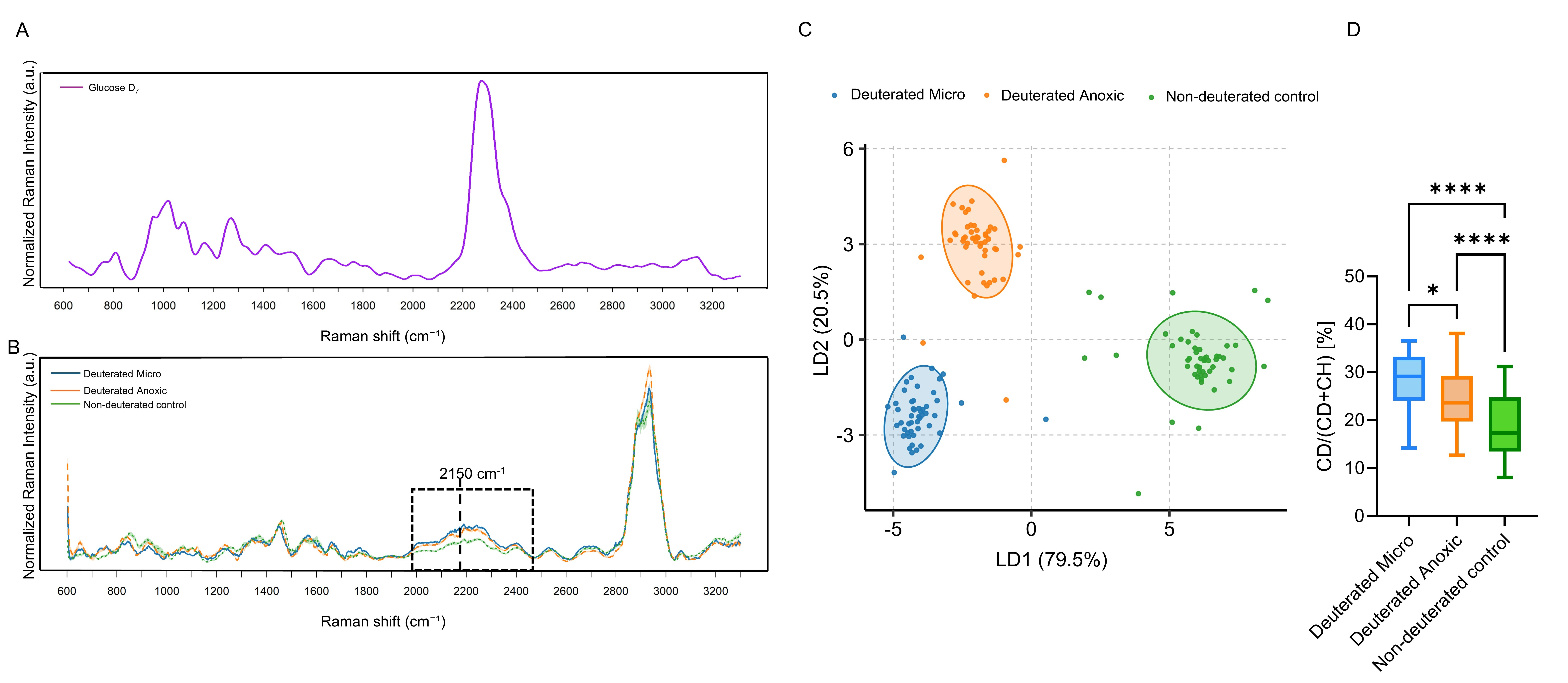


**Figure S5:** Detection of glucose uptake in TM7x by Raman spectroscopy coupled with glucose-d_7_ labelling. (A) Raman spectrum of glucose-d_7_, showing the C–D bond-specific peak at ~2115 cm⁻¹. (B) Raman spectra of TM7x grown in micro-oxic (blue) or anoxic (brown) or unlabelled (green) control conditions with glucose-d7, both showing incorporation of glucose as evidenced by the 2115 cm⁻¹ peak. (C) Linear discriminant analysis (LDA) plot illustrating clear separation among deuterated micro-oxic, deuterated anoxic, and unlabeled control groups (PERMANOVA: 0.47, *P* = 0.001). (D) Quantification of the C–D/(C–D + C–H) signal ratio demonstrating significantly higher deuterium incorporation in glucose-d_7_–treated samples compared to unlabeled controls.

| **Supplemental Table 1**: Raman peak assignments and arginine-dependent spectral changes | | | |  |
| --- | --- | --- | --- | --- |
|  |  |  |  |  |
| Raman peak (cm^-1^) | Potential Raman peak assignments | Micro-oxic |  |  |
| 635 | Tyrosine [29] | Increasing |  |  |
| 780 | Nucleic Acids [28] | Stable |  |  |
| 1003 | Phenylalanine [23, 24, 25] | Increasing |  |  |
| 1450 | Lipids, proteins (CH₂ bending) [23, 24, 25] | Stable |  |  |
| 1606 | Tyrosine, phenylalanine [29] | Increasing |  |  |
| 1660 | Lipids, proteins, amide I [23, 24, 25] | Increasing |  |  |
| 1730 | Lipids (ester C=O) [30] | Stable |  |  |
|  |  |  |  |  |
| Stable = no significant change, Increasing = higher intensity in glucose-plus, Decreasing = lower  intensity in glucose-plus | | | | |

| **Supplemental Table 2:** Raman peak assignments and glucose-dependent spectral changes | | | |
| --- | --- | --- | --- |
|  |  |  |  |
|  |  |  |  |
| Raman peak (cm^-1^) | Potential Raman peak assignments | Micro-oxic | Anoxic |
|  |  |  |  |
| 635 | Tyrosine [29] | Stable | Increasing |
| 780 | Nucleic Acids [28] | Increasing | Increasing |
| 1003 | Phenylalanine [23, 24, 25] | Increasing | Decreasing |
| 1450 | Lipids, proteins (CH₂ bending) [23, 24, 25] | Stable | Decreasing |
| 1606 | Tyrosine, phenylalanine [29] | Stable | Stable |
| 1660 | Lipids, proteins, amide I [23, 24, 25] | Stable | Stable |
| 1730 | Lipids (ester C=O) [30] | Stable | Increasing |
|  |  |  |  |
| Stable = no significant change, Increasing = higher intensity in glucose-plus, Decreasing = lower intensity in glucose-plus | | | |

| **Supplemental Table 3:** Primers used in this study | |  |
| --- | --- | --- |
|  |  |  |
| Primer Name | 5'-3' | Tm |
| ArcA_up_fwd | GCACCATATGCAGTACCTTAGCCGTATC | 62 |
| ArcA_Up_rev | ACTGCTGGTCATTTTAATGGCTCAGAGTTTG | 70 |
| HphI_ArcA_fwd | CCATTAAAATGACCAGCAGTGTTTACATAATTG | 66 |
| HphI_ArcA_rev | GAGGGTTGTGATAATACAACCAAAATACCCC | 69 |
| ArcA-Down_fwd | GTTGTATTATCACAACCCTCCCCTGTTTATATTAC | 68 |
| ArcA_dwn_rev | GCTCGAATTCTTCTCCTAATGACGTGCC | 61 |
| ArcA+HphI_upstream | GCTTCGCCAAATCTTCCAC | 64 |
| ArcA+HphI_downstream | CCTTCATTCAGAATTTCAAAAAGG | 60 |
| ArcA_PCWU3_fwd | ATTAGGAGAAGAATTCGAGCTCGGTACC | 69 |
| ArcA_PCWU3_rev | TAAGGTACTGCATATGGTGCACTCTCAG | 70 |
| ArcE_up_fwd | GCACCATATGATCATGCAGAGCTTGCTG | 72 |
| ArcE-Up_rev | ACTGCTGGTCCACATGGATAAGAAGCGTAC | 73 |
| HphI_ArcE_fwd | ATCCATGTGGACCAGCAGTGTTTACATAATTG | 70 |
| HphI_ArcE_rev | GGTAGAAAAAATAATACAACCAAAATACCCC | 64 |
| ArcE-down_fwd | GTTGTATTATTTTTTCTACCATTGCCTCG | 64 |
| ArcE_down_rev | GCTCGAATTCCAATTGCGCCACTTCCAG | 74 |
| pCWU3_ArcE_fwd | GGCGCAATTGGAATTCGAGCTCGGTACC | 76 |
| pCWU3_ArcE_rev | TCTGCATGATCATATGGTGCACTCTCAG | 70 |
| ArcE+HphI_upstream | CGCTTAAGTCAACAATTGTCTG | 62 |
| ArcE+HphI_downstream | GTGCTGACGTGATTTACAC | 61 |
| ASG_094 | ATAGCGATGACAGCTACGCC | 67 |
| ASG_095 | GCGTCTCTTCAAAAACCTCGAAAT | 65 |
| Hyg_F_check | CTCCTGGTCATTGCGCAGTG | 69 |
| Hyg_R_check | CTCTCAGGCTTCACAGACC | 65 |
| ASG_102 | TCGAAGAGCAGAAACGCACA | 67 |
| ASG_103 | TCTTTTGCCTTCGCCTCAGT | 67 |
| 16S F | GGCCTTCGGGTTGTAAAC | 64 |
| 16S R | CTGCTGGCACGTAGTTAG | 63 |
| Ldh_F | CTTTGCTATCGACGTTTAG | 57 |
| Ldh_R | GTATTTGGTGGTCGTCAG | 60 |
| TpiA_F | CACTAGTCAGCTGATCCG | 62 |
| TpiA_R | GATATCCGGTTGAAAGTTC | 57 |
| GPD_F | CTAGCGGTGTAGCTATGC | 62 |
| GPD_R | CAACGCCAATTGTATCTAAC | 58 |
| PFL_F | GACATCAAAACCAAGACC | 58 |
| PFK_F | GAAGCATCGGCATATTACC | 60 |
| PFK_R | CGCATTTAGTACGATATTCCC | 60 |
| TKT_1_F | CTTCCGCCGTAATAACAG | 60 |
| TKT_1_R | CAACTGTTATTAGCGGCAG | 61 |
| ArgR F | GGCTCTCATCTATACAGTG | 58 |
| ArgR R | GGTACGCAGGACGAGCTG | 69 |
| ArcB F | GCTTCGCCAAATCTTCCAC | 64 |
| ArcB R | GAAGATACGGCTAAGGTAC | 59 |
| ArcC F | CGCTTAAGTCAACAATTGTCTG | 62 |
| ArcC R | CATGAAGAGGCGATTAACAC | 61 |
| ArgG_F | GAAGACCTTGAGGTCATTC | 60 |
| ArgG_R | GTACTTGCCTTCGTACAG | 60 |
| NS1up_GFP_F | AAATTGCTGCGGCGACTC | 67 |
| NS1up_GFP_R | CTGATGGTTGTGTTTGCTCCATAGATGCG | 71 |
| sfGFP+HphII_F | GGAGCAAACACAACCATCAGCTATTTACAAC | 69 |
| sfGFP+HphII_R | CTCACTTGTCTTAGCCAAACAAAAATCCC | 68 |
| NS1Dwn_GFP_F | GTTTGGCTAAGACAAGTGAGAGAATCTG | 67 |
| NS1Dwn_GFP_R | AATCAATGAATACCTTATGAAAG | 55 |
| NS1up_Pro_F | AAATTGCTGCGGCGACTC | 67 |
| NS1up_Pro_R | TGCTCACCATAGAGGTATTTCTCCTAACTTAATATTGATGATATTC | 69 |
| mNeonGreen(t)_F | AAATACCTCTATGGTGAGCAAGGGCGAG | 72 |
| mNeonGreen(t)_R | TAGCGATCGGTTACTTGTACAGCTCGTCCATG | 74 |
| Term_NS1dwn_F | GTACAAGTAACCGATCGCTACTTTTGCAAC | 69 |
| Term_NS1dwn_R | AATCAATGAATACCTTATGAAAGGTAAG | 60 |
| pArcA_F | TCTACTCTTGCCCAAAAACATAAAC | 63 |
| pArcA_R | TTGCTCACCATCACAACCCTCCCCTGTTTATATTAC | 73 |
| mNG(a)_F | GGAGGGTTGTGATGGTGAGCAAGGGCGAG | 79 |
| mNG(a)_R | GACAACAACTATTACTTGTACAGCTCGTCCATG | 70 |
| ArcAterm_F | TGTACAAGTAATAGTTGTTGTCGTAATAAGG | 64 |
| ArcAterm_R | GCTTTGCTTTTTTAATTTTTTCTAC | 57 |
| pTuf_Backbone_F | ATTCATTGATGAATTCGAGCTCGGTACC | 68 |
| pTuf_Backbone_R | CGCAGCAATTCATATGGTGCACTCTCAG | 72 |
| TM7x_pTuf_NS1_mNG_F | GCACCATATGAATTGCTGCGGCGACTCTG | 75 |
| TM7x_pTuf_NS1_mNG_R | GCTCGAATTCATCAATGAATACCTTATGAAAGGTAAGCTTC | 70 |
| pArcA_Backbone_F | TGGAAACACTGAATTCGAGCTCGGTACC | 72 |
| pArcA_Backbone_R | CATTGTTATTCATATGGTGCACTCTCAG | 65 |
| TM7x_pArcA_NS1_mNG_F | GCACCATATGAATAACAATGAACGAGCTGAATACG | 70 |
| TM7x_pArcA_NS1_mNG_R | GCTCGAATTCAGTGTTTCCAACACGGGC | 74 |
| Up_pArc_mNG_F | GCACCATATGAATTGCTGCGGCGACTCTG | 75 |
| Up_pArc_mNG_R | ACTGCTGGTCCTATTACTTGTACAGCTCGTCCATG | 75 |
| pTuf_Hyg_Term_Dwn_F | CAAGTAATAGGACCAGCAGTGTTTACATAATTG | 67 |
| pTuf_Hyg_Term_Dwn_R | GCTCGAATTCATCAATGAATACCTTATGAAAGG | 67 |
| NeonHygro_Backbone_F | ATTCATTGATGAATTCGAGCTCGGTACC | 68 |
| NeonHygro_Backbone_R | CGCAGCAATTCATATGGTGCACTCTCAG | 72 |
